# Effect of itraconazole on pharmacokinetics of ZX-7101A tablets in healthy Chinese subjects

**DOI:** 10.3389/fphar.2025.1722747

**Published:** 2025-12-18

**Authors:** Junzhen Wu, Yin Wang, Wei Liu, Jinjie He, Jufang Wu, Jicheng Yu, Xiaojie Wu, Jianguang Su, Mei Liu, Yilin Li, Jing Zhang

**Affiliations:** 1 Clinical Pharmacology Research Center, Huashan Hospital, Fudan University, Shanghai, China; 2 Nanjing Zenshine Pharmaceuticals Co., Ltd, Nanjing, China; 3 Department of Clinical Nutrition, Huashan Hospital, Fudan University, Shanghai, China; 4 Institute of Antibiotics, Huashan Hospital, Fudan University, Shanghai, China; 5 Key Laboratory of Antibiotic Clinical Pharmacology of the National Health and Commission, Shanghai, China; 6 National Clinical Research Center for Aging and Medicine, Huashan Hospital, Fudan University, Shanghai, China; 7 Research Ward of Huashan Hospital, Fudan University, Shanghai, China

**Keywords:** drug-drug interaction, itraconazole capsule, pharmacokinetics, ZX-7101, ZX-7101A tablet

## Abstract

**Aims:**

A single-center, open label trial was conducted to evaluate the effects of itraconazole on the pharmacokinetics of ZX-7101A tablets in healthy Chinese adults.

**Methods:**

Subjects took a single dose of 40 mg ZX-7101A tablets on Day 1 on Day 31following once-daily itraconazole administration (200 mg, Days 26–50). The concentrations of ZX-7101A and ZX-7101 in plasma samples were determined by liquid chromatography–tandem mass spectrometry. Pharmacokinetic (PK) parameters of ZX-7101A and ZX-7101 were calculated, and the effects of itraconazole on the PK of ZX-7101 were evaluated.

**Results:**

The median T_max_ of ZX-7101 was 4 h. In stage 1, the mean (±SD) C_max_, AUC_0-t_, AUC_0-inf_, and t_1/2_ of ZX-7101 were 90.86 ± 44.48 ng/mL, 6313.72 ± 1095.17 h*ng/mL, 6827.31 ± 1163.30 h*ng/mL, and 126.40 ± 28.88 h, respectively. In stage 2, the corresponding values were 117.63 ± 29.95 ng/mL, 9706.83 ± 1062.56 h*ng/mL, 10785.99 ± 1389.08 h*ng/mL, and 148.62 ± 28.72 h. Itraconazole increased ZX-7101 C_max_, AUC_0-t_, and AUC_0-inf_ by 29.5%, 53.7%, and 58.0%, respectively, and prolonged t_1/2_ of ZX-7101 by 17.6%.

**Conclusion:**

The ZX-7101 exposure after coadministration with itraconazole is lower than the exposure after a single dose of 80 mg ZX-7101A tablets. It is therefore not necessary to adjust the dose of ZX-7101A 40 mg when coadministered with itraconazole.

**Trial registration:**

http://www.chinadrugtrials.org.cn, identifier: CTR20231556.

## 1 Introduction

Influenza A and B viruses can cause serious and highly contagious infections, and induce life-threatening complications, especially in high-risk patients such as the elderly or immunocompromised populations (Eichberg et al., 2022). Influenza epidemic therefore imposes a great burden on human health, society, and economy. In addition to vaccines, antiviral drugs are important interventions for controlling and managing the disease in order to reduce the mortality rate of patients or control regional pandemics (Shirley, 2020). Baloxavir marboxil is a novel Cap-dependent endonuclease (CEN) inhibitor targeting the PA protein subunit of influenza virus polymerase complex. A meta-analysis showed that baloxavir marboxil has better safety and more potent efficacy than oseltamivir, and tends to shorten the course of disease (Shiraishi et al., 2024). Meanwhile, this class of drugs has made breakthrough progress in treating oseltamivir-resistant influenza strains (Heo, 2018). ZX-7101A is a highly effective, orally administered CEN inhibitor developed by Nanjing Zenshine Pharmaceuticals Co., Ltd. ZX-7101A is a prodrug that is transformed to the active metabolite ZX-7101 (parent drug) via lipase-catalyzed hydrolysis in body to exert antiviral activity. *In vitro* enzyme activity assays demonstrated high inhibitory activity of ZX-7101 against influenza virus CEN (IC_50_ = 21.7 nM), nearly comparable to that of baloxavir marboxil (IC_50_ = 14.8 nM).

The data from a Phase I clinical trial indicate that ZX-7101A is generally safe and well-tolerated in healthy subjects (Wu et al., 2024). ZX-7101A was rapidly transformed to the active ingredient ZX-7101 in healthy subjects after a single oral dose of 40 mg–320 mg. The blood concentration of ZX-7101A was below the lower limit of quantification (LLOQ) at most of the time points after administration. ZX-7101 reaches its peak concentration 3–4 h after administration, with t_1/2_ ranging from 83.01 h to 125.55 h. Within the dose range from 40 mg to 320 mg, the exposure (C_max_ and AUC) of ZX-7101 increased slightly less than proportional to the dose. The C_max_ of ZX-7101 decreased by about 43.35% after a single oral dose of 80 mg ZX-7101A under fed condition compared to fasting conditions in healthy subjects, while the AUC of ZX-7101 decreased by about 33.23%–34.48% compared to the corresponding values under fasting condition. However, postprandial administration did not affect the T_max_ and t_1/2_ of ZX-7101. Single oral doses of 40 mg and 80 mg of ZX-7101A tablets were selected for Phase II/III clinical studies in adults with uncomplicated influenza. The results of the Phase II/III study demonstrated that a single dose of 40 mg or 80 mg ZX-7101A was effective in treating uncomplicated influenza in adults with safety profiles similar to that of placebo (Wang et al., 2025).

Preclinical metabolic studies indicate that prodrug ZX-7101A is transformed to ZX-7101 via recombinant carboxylesterases (CES) CES1-b, CES1-c, and CES2. CYP450 enzymes are not involved in the metabolism of ZX-7101A and ZX-7101. Neither ZX-7101A nor ZX-7101 has a significant inhibitory or inducing effect on CYP450 metabolic enzymes. Caco-2 cell transport studies demonstrated that both ZX-7101A and its metabolite ZX-7101 exhibited pronounced basolateral-to-apical efflux in the absence of the P-gp inhibitor verapamil. For ZX-7101 at 10 μM, the efflux ratio was 13.8, which decreased to 2.43 with verapamil (82.4% reduction). For ZX-7101A, efflux ratios were 10.8 at 2 μM and 7.18 at 20 μM, which dropped to 1.19 (89.0% reduction) and 1.20 (83.3% reduction), respectively, upon verapamil treatment. In contrast, co-incubation with the breast cancer resistance protein (BCRP) inhibitor novobiocin produced negligible changes in efflux ratios for either compound, with differences ranging from −15.2% to +3.01% across the concentrations tested, indicating no meaningful interaction with BCRP. These findings indicate that ZX-7101 and ZX-7101A are substrates of P-gp but not BCRP (unpublished data). According to the International Conference on Harmonization (ICH) M12 guidelines, itraconazole is a clinically relevant P-gp inhibitor commonly used in clinical DDI studies (ICH, 2022). This study was designed to assess the effect of multiple oral doses of itraconazole capsules (P-gp inhibitor) on the pharmacokinetic (PK) profiles of ZX-7101A and its active metabolite ZX-7101 after a single oral dose of ZX-7101A tablets in healthy Chinese adults, and evaluate the safety and tolerability of ZX-7101A monotherapy and ZX-7101A/itraconazole combination therapy. The findings will provide critical insights into drug-drug interactions, supporting the clinical development of ZX-7101A.

## 2 Materials and methods

### 2.1 Study design

This single center, open label, fixed sequence trial consisted of two stages. Stage 1 was single oral administration of ZX-7101A tablets to the subjects under fasting condition, while Stage 2 included coadministration of ZX-7101A tablets and itraconazole capsules. Participants took ZX-7101A tablets 40 mg orally under fasting condition in the morning on D1 of Stage 1 and D31 of Stage 2. In drug-drug interaction clinical trials, the selected dose of the precipitant drug should maximize the potential to identify drug-drug interaction in the context of safety (i.e., achieving an exposure level close to the highest clinically recommended dose). Thus, the clinically recommended maximum dose and the shortest dosing interval of the precipitant drug should generally be evaluated (ICH, 2022). The PK of itraconazole is influenced by its formulation, food intake, and gastrointestinal pH (Liu et al., 2016; Allen et al., 2015). For itraconazole capsules, oral administration of 200 mg itraconazole after meals results in a plasma exposure 1.6 times higher than those under fasting condition (Liu et al., 2016). Therefore, in this study, itraconazole was administered orally after meals to achieve maximum clinical exposure. Therefore, from D26 to D50 of Stage 2, the participants took 200 mg itraconazole capsules once daily for 25 consecutive days within 30 min after eating a standard breakfast (coadministration with ZX-7101A at the same time in the morning of D31 under fasting condition). The subjects were discharged from the study after completing all the examinations on D50. It was planned to enroll 16 subjects to ensure that at least 12 subjects could complete the trial.

### 2.2 Investigational product

ZX-7101A (40 mg/tablet) was provided by Nanjing Zenshine Pharmaceuticals Co., Ltd. Itraconazole capsules (100 mg/capsule) were purchased from Xi’an Janssen Pharmaceutical Ltd.

### 2.3 Study participants and ethical approval

This clinical trial was reviewed and approved by the Institutional Review Board of Huashan Hospital, Fudan University (approval No.: 2023–556). All subjects signed their informed consent forms before screening.

The enrolled participants were healthy males or females aged 18–45 years with body mass index (BMI) of 19–28 kg/m^2^. Medical history, physical examination, vital signs, 12-lead electrocardiography (ECG), laboratory tests, and lung computed tomography did not indicate any abnormality of clinical significance. All participants agreed to take contraceptive measures and had no plans to conceive or donate sperm/eggs from signature of informed consent form until 6 months (females) or 90 days (males) after the last dose of the investigational product.

Subjects with any of the following conditions were excluded: a clinically significant history of disease, a possible history of allergies to the investigational products, or a history of allergies to two or more other foods or drugs. Individuals who had used any P-gp or CYP inducers or inhibitors within 30 days, or any prescription drugs or herbal medicines within 1 month, or any health supplements within 2 weeks, were excluded. Subjects who were found with alcoholism or smoking more than 5 cigarettes per day were excluded. Individuals with a history of drug abuse or with a positive result in drug abuse screening test were excluded. Pregnant or lactating women or those with positive blood pregnancy test were excluded.

### 2.4 PK sample collection and assay

In Stage 1, blood samples were collected 2 h pre-dose on D1, and 1, 2, 3, 4, 5, 6, 8, 12, 24 h (D2), 48 h (D3), 96 h (D5), 144 h (D7), 216 h (D10), 336 h (D15), and 456 h (D20) post-dose. In Stage 2: blood samples were collected 2 h pre-dose on D31, and 1, 2, 3, 4, 5, 6, 8, 12, 24 h (D32), 48 h (D33), 96 h (D35), 144 h (D37), 216 h (D40), 336 h (D45), and 456 h (D50) post-dose. The samples were collected in tubes containing heparin sodium and dichlorvos and centrifuged to prepare plasma. A fully validated analytical method of liquid chromatography–tandem mass spectrometry (LC-MS/MS) was used to determine the concentrations of ZX-7101A and ZX-7101 in the human plasma samples. Baloxavir was used as the internal standard. The linear range was 0.2–200 ng/mL for both ZX-7101A and ZX-7101.

### 2.5 Calculation of PK parameters

Pharmacokinetic analyses were performed using Phoenix WinNonlin (version 8.3.1 or higher, Pharsight Corporation, Mountain View, CA, United States). Noncompartmental analysis (NCA) was applied to derive PK parameters based on actual sampling times and measured plasma concentrations. Concentrations below the lower limit of quantification (BLQ) occurring before Tmax were set to zero, whereas BLQ values occurring after Tmax were treated as “not detected” (ND). For descriptive statistics, BLQ values were excluded from analysis; sampling time points with BLQ values in more than one-third of subjects were not summarized and were reported as “NC.” AUC_0–t_ was calculated using the linear-up/log-down trapezoidal method. The terminal elimination rate constant (λ_z_) was estimated by linear regression of the log-linear terminal phase, and the elimination half-life (t_1/2_) was calculated as ln (2)/λ_z_. AUC_0–inf_ was calculated as AUC_0–t_ plus the extrapolated portion (C_t_/λ_z_). PK parameters dependent on λ_z_ (including λ_z_, t_1/2_, CL/F, Vz/F, and AUC_0–inf_) were excluded from analysis when the extrapolated portion of AUC_0–inf_ exceeded 20% (i.e., AUC_0-t_/AUC_0–inf_ < 0.8) or when fewer than three quantifiable data points were available for λ_z_ estimation. Missing PK data were flagged as “Missing” and were not included in any analysis. Any outliers identified during statistical review were only excluded after joint evaluation by the sponsor and investigators.

### 2.6 Drug-drug interaction analysis

The primary PK parameters of ZX-7101 (C_max_, AUC_0-t_, and AUC_0-inf_) after administration of ZX-7101A alone and in combination with itraconazole were log-transformed and analyzed by analysis of variance (ANOVA). The geometric mean ratios of PK parameters and the corresponding 90% confidence intervals (CIs) were calculated. It was determined that multiple doses of itraconazole capsules have no effect on the PK characteristics of ZX-7101 after a single dose of ZX-7101A tablets if the 90% CI falls within 80.00%–125.00%.

### 2.7 Safety assessment

The safety of subjects was assessed in terms of adverse events (AEs) and serious adverse events (SAEs) after administration based on the findings of physical examination, vital signs, ECG, and laboratory tests.

### 2.8 Statistical analysis

Statistical software SAS (v9.4, SAS Institute Inc., North Carolina, United States) and Phoenix WinNonlin (v8.3.1, Pharsight Corp, Mountain View, CA, United States) were used to calculate and analyze the baseline demographic data and PK parameters.

## 3 Results

### 3.1 Demographic data and disposition of subjects

Overall, 78 of the 94 subjects who signed informed consent forms failed in screening. Finally, 16 subjects were enrolled. Two subjects withdrew from the trial early at Stage 1. The remaining 14 subjects completed the trial as per protocol. The mean age and body weight of the 16 participants was 27.88 ± 6.01 years old and 63.01 ± 7.76 kg, respectively (Table 1).

**TABLE 1:** Demographic and baseline characteristics of participants in the drug-drug interaction study.

Characteristic	Statistics (*n* = 16)
Age (years)	27.88 ± 6.01
Height (cm)	165.27 ± 8.78
Body weight (kg)	63.01 ± 7.76
Body mass index (kg/m^2^)	23.09 ± 2.48
Sex	
Male	10 (62.5)
Female	6 (37.5)
Race	
Han	15 (93.8)
Others	1 (6.3)

Data are presented as mean ± SD, or number (%) unless otherwise specified.

### 3.2 PK parameters of ZX-7101

Overall, 16 subjects were included in the PK parameter set (PKPS). Subject R004 withdrew early at Stage 1 after PK blood samples were collected at 216 h (D10) post-dose, while subject R007 withdrew early at Stage 1 after PK blood sample was collected at 96 h (D5) post-dose. All other subjects completed PK blood sample collection according to the protocol.

**FIGURE 1:**
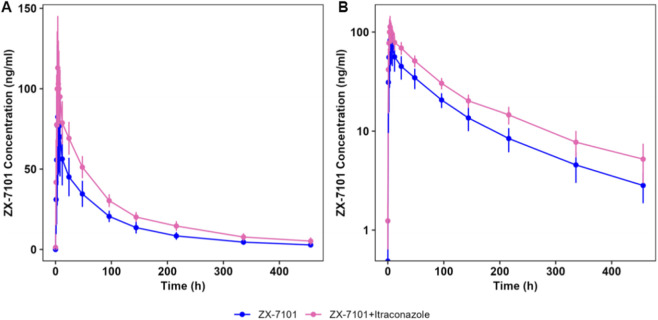
ZX-7101 plasma concentration-time profiles following a single oral dose of 40 mg ZX-7101A tablets in healthy Chinese subjects with or without coadministration of multiple oral doses of 200 mg itraconazole. **(A)** Linear scale. **(B)** Semi-logarithmic scale. Data are illustrated as mean ± standard deviation.

Plasma concentration of ZX-7101A was below the LLOQ at most time points after a single oral dose of 40 mg ZX-7101A tablets alone or in combination with itraconazole under fasting condition in most of the subjects in PKPS. ZX-7101A was detectable only at a few time points, making it impossible to calculate the PK parameters of ZX-7101A. Therefore, descriptive statistical analysis was not performed on the PK parameters of ZX-7101A. The prodrug ZX-7101A was rapidly transformed to the parent drug ZX-7101 *in vivo*. Figure 1 illustrates the plasma concentration-time curves of ZX-7101 during single dose ZX-7101A study and ZX-7101A/itraconazole combination therapy. After a single dose of 40 mg ZX-7101A tablets alone or in combination with itraconazole in the healthy subjects, the median T_max_ of ZX-7101 was 4 h. The mean plasma C_max_ was 90.86 ± 44.48 and 117.63 ± 29.95 ng/mL, respectively. The corresponding AUC_0-t_ was 6313.72 ± 1095.17 and 9706.83 ± 1062.56 h*ng/mL, respectively. AUC_0-inf_ was 6827.31 ± 1163.30 and 10785.99 ± 1389.08 h*ng/mL, respectively. Plasma t_1/2_ was 126.40 ± 28.88 and 148.62 ± 28.72 h (Table 2). These results indicated that coadministration with itraconazole did not affect the absorption rate of ZX-7101A significantly, but increased the exposure of ZX-7101, evidenced by average increase of C_max_, AUC_0-tt_, and AUC_0-inf_ by 29.5%, 53.7%, and 58.0%, respectively. Itraconazole also prolonged t_1/2_ of ZX-7101 slightly by 17.6%.

**TABLE 2:** Pharmacokinetic parameters of ZX-7101 after single oral dose administration of 40 mg ZX-7101A alone or combined with multiple oral doses of 200 mg itraconazole in healthy subjects.

Parameter	ZX-7101A alone (*n* = 16)	ZX-7101A + itraconazole(*n* = 14)
t_1/2_ (h)	126.40 ± 28.88	148.62 ± 28.72
T_max_ (h)	4 (1, 6)	4 (2, 8)
C_max_ (ng/mL)	90.86 ± 44.48	117.63 ± 29.95
AUC_0-t_ (h*ng/mL)	6313.72 ± 1095.17	9706.83 ± 1062.56
AUC_0-inf_ (h*ng/mL)	6827.31 ± 1163.30	10785.99 ± 1389.08
k_e_ (1/h*10^–3^)	5.90 ± 2.05	4.83 ± 0.95

t_1/2_, half-life; T_max_, time to maximum concentration; C_max_, maximum concentration; AUC_0-t_, the area under the plasma concentration-time curve from time 0 to t; AUC_inf_, the area under the plasma concentration-time curve from time 0 to infinity; k_e_, elimination rate constant.

### 3.3 Drug-drug interaction

Table 3 presents the geometric mean ratio of exposure PK parameters of ZX-7101 and the corresponding 90% CI between ZX-7101A tablets administered alone and in combination with itraconazole. The geometric mean ratio of ZX-7101 C_max_ was 1.38 (90% CI: 1.10, 1.73). The lower limit of 90% CI falls within the range of 0.8–1.25. The geometric mean ratio of AUC_0-t_ and AUC_0-inf_ was 1.56 (90% CI: 1.41, 1.73) and 1.59 (90% CI: 1.42, 1.78), respectively. The upper and lower limits of the 90% CIs are all outside the range of 0.8–1.25. The results indicated that multiple doses of itraconazole capsules may increase the exposure of the active metabolite ZX-7101 in human body after a single dose of 40 mg ZX-7101A tablets.

**TABLE 3:** Geometric mean ratio of pharmacokinetic parameter of ZX-7101 after a single oral dose of 40 mg ZX-7101A with and without coadministration of multiple oral doses of 200 mg itraconazole.

PK parameter of ZX-7101	(ZX-7101A + Itraconazole) / ZX-7101A alone	
	GMR	90% CI
C_max_	1.38	(1.10,1.73)
AUC_0–456h_	1.56	(1.41,1.73)
AUC_0-inf_	1.59	(1.42,1.78)

PK, pharmacokinetic; GMR, geometric mean ratio; CI, confidence interval; C_max_, maximum plasma concentration; AUC_0-inf_, the area under the plasma concentration-time curve from time 0 to infinity.

### 3.4 Safety and tolerability

Overall, 13 subjects (81.3%, 13/16) experienced 33 AEs, all of which were treatment emergent AEs (TEAEs) (Table 4). In the first stage of study (single dose of ZX-7101A tablets alone), four subjects (25.0%, 4/16) experienced a total of 6 TEAEs, all of which were Common Terminology Criteria for Adverse Events (CTCAE) Grade 1. During administration of itraconazole capsule monotherapy, a total of 4 TEAEs occurred in three subjects (21.4%, 3/14). All the AEs were Grade 1 except for rash, which was Grade 2. During coadministration of ZX-7101A/itraconazole and follow-up period, nine subjects (64.3%, 9/14) experienced a total of 23 TEAEs, most of which were Grade 1 or 2 in severity. One subject experienced an elevation in creatine kinase.

**TABLE 4:** Treatment-emergent adverse events that occurred in at least 1 subject.

TEAE	D1-D25 n (%)	D26-D30 n (%)	D31-D50 n (%)	D51 to the end of study n (%)
	ZX-7101A	Itraconazole	ZX-7101A +Itraconazole	​
	(N = 16)	(N = 14)	(N = 14)	(N = 14)
Subjects with at least one TEAE	4 (25.0)	3 (21.4)	9 (64.3)	1 (7.1)
Infectious and infestations	1 (6.3)	0	1 (7.1)	1 (7.1)
COVID-19	1 (6.3)	0	0	0
Upper respiratory tract infection	0	0	1 (7.1)	1 (7.1)
Investigations	1 (6.3)	0	4 (28.6)	1 (7.1)
Conjugated bilirubin increased	0	0	1 (7.1)	0
Protein in urine	1 (6.3)	0	0	0
Urinary occult blood positive	1 (6.3)	0	1 (7.1)	0
Aspartate aminotransferase increased	0	0	0	1 (7.1)
Total bilirubin increased	0	0	2 (14.3)	0
Myoglobin increased	0	0	0	1 (7.1)
Creatine kinase elevation	0	0	1 (7.1)	1 (7.1)
Lactic dehydrogenase increased	0	0	0	1 (7.1)
Respiratory, thoracic and mediastinal disorders	0	1 (7.1)	1 (7.1)	0
Oropharyngeal pain	0	1 (7.1)	1 (7.1)	0
Skin and subcutaneous tissue disorders	0	1 (7.1)	0	1 (7.1)
Rash	0	1 (7.1)	0	1 (7.1)
General disorders and administration site conditions	1 (6.3)	0	0	0
Chest discomfort	1 (6.3)	0	0	0
Reproductive system and breast disorders	0	0	2 (14.3)	0
Menstruation irregular	0	0	2 (14.3)	0
Gastrointestinal disorders	0	0	3 (21.4)	0
Nausea	0	0	1 (7.1)	0
Abdominal discomfort	0	0	1 (7.1)	0
Mouth ulceration	0	0	2 (14.3)	0
Toothache	0	0	1 (7.1)	0
Cardiac disorders	1 (6.3)	1 (7.1)	1 (7.1)	0
Nodal rhythm	0	0	1 (7.1)	0
First degree atrioventricular block	1 (6.3)	1 (7.1)	0	0

Data are presented as number (%) unless otherwise specified. TEAE, treatment-emergent adverse event; COVID-19, infectious disease caused by the SARS-CoV-2, coronavirus.

None of the subjects developed any drug-related TEAE while ZX-7101A or itraconazole was administered alone. During itraconazole/ZX-7101A coadministration and follow-up period, three subjects (21.4%, 3/14) developed 10 cases of ZX-7101A-related TEAEs. Four subjects (28.6%, 4/14) developed 11 cases of itraconazole-related TEAEs. One subject developed creatine kinase elevation, which was evaluated by the investigator as SAE. All other TEAEs were classified as Grade 1 or 2. The adverse drug reactions (ADRs) associated with ZX-7101A included elevated creatine kinase, increased aspartate transaminase (AST), increased myoglobin, increased lactate dehydrogenase (LDH), nausea, gastric discomfort, and elevated total bilirubin. The ADRs of itraconazole were creatine kinase elevation, AST increased, myoglobin increased, LDH increased, total bilirubin increased, conjugated bilirubin increased, and irregular menstruation.

During the follow-up period after itraconazole/ZX-7101A coadministration, one case of SAE occurred, which was a CTCAE Grade 4 creatine kinase elevation. The subject showed a mild elevation of creatine kinase (CTCAE Grade 2, 1065 U/L, normal range: 50–310 U/L) on the last day of Stage 2 (D50), which was the 20th day after the second dose of ZX-7101A and the 25th day after the first dose of itraconazole. The subject reported no discomfort and had no history of vigorous exercise or took other medications. But subsequent testing indicated a further elevation in creatine kinase, reaching CTCAE Grade 3 (2166 U/L), and later Grade 4 (3231 U/L). The subject received therapies including hydration, alkalization, and liver function protection. Further monitoring showed persistent increase of creatine kinase (7084 U/L on D62), accompanied by elevated serum levels of myoglobin, AST, and LDH. Further investigations, including autoimmune antibodies tests, thyroid function tests, magnetic resonance (MR) imaging of the thigh, and electromyography, revealed no abnormalities. This AE was reported as SAE based on “other important medical events”. The level of creatine kinase was ultimately normalized on Day 80. Considering the temporal relationship between the occurrence of this AE and the administration of ZX-7101A and itraconazole, as well as the absence of other factors that could explain the elevation of creatine kinase, the investigator concluded that this SAE was possibly related to itraconazole but not to ZX-7101A.

## 4 Discussion

In animal studies, the absolute bioavailability of ZX-7101A was lower than 30% in rats and dogs (unpublished data). *In vitro* experiments revealed that both ZX-7101 and ZX-7101A are substrates of P-gp transporters. Given these preclinical data, the PK profile of ZX-7101A and ZX-7101 could be altered by coadministered inhibitors of P-gp transporters. This study assessed the effects of itraconazole, a clinically relevant P-gp inhibitor, on the PK profile of ZX-7101A and ZX-7101 after a single oral dose of 40 mg ZX-7101A tablets in healthy Chinese subjects. The findings provide support for possible concomitant use of ZX-7101A tablet and drugs inhibiting P-gp transporter in clinical practice.

ZX-7101A showed good tolerability within the dose range from 40 mg to 320 mg in a phase I clinical trial (Wu et al., 2024). The results of a Phase II/III study demonstrated that a single dose of ZX-7101A 40 mg or 80 mg was effective in treating uncomplicated influenza in adults with placebo-like safety profiles (Wang et al., 2025). Therefore, 40 mg was proposed as the dose used in this study.

P-gp is widely expressed in various tissues, especially on the basal and apical surfaces of intestinal epithelial cells, as well as in the bile ducts and proximal tubules of the kidney (Fromm, 2004). P-gp in the gastrointestinal tract affects drug absorption and has a significant impact on PK of drugs, such as inhibiting the bioavailability of digoxin, paclitaxel, cyclosporine, and HIV protease inhibitors (Lee et al., 2010). In addition to intestinal epithelial cells, P-gp is also highly expressed in the bile ducts of liver cells, where it functions to transport substrates and other compounds from liver cells into bile (Liu, 2019). Consequently, drugs that are not metabolized and mainly excreted through bile are susceptible to drug interactions. Additionally, the kidney, being an important excretory organ, relies on P-gp in the proximal tubules to facilitate the excretion of drugs and their metabolites into urine (Lee et al., 2010). The preclinical experiments in rats using radio-labelled ZX-7101A demonstrated that ZX-7101A and its active metabolite ZX-7101 were mainly excreted in feces and bile, with only a very small fraction excreted through urine (unpublished data). The clinical mass balance study of ZX-7101A using radio-labelled ZX-7101A also found that both ZX-7101A and its active metabolite ZX-7101 were primarily excreted in feces and bile, with minimal urinary excretion (unpublished data). This study found that concomitant use of itraconazole increased the exposure level (C_max_, AUC) of ZX-7101 by nearly 1.5 times, but had no significant effect on the absorption rate of ZX-7101A. These findings suggest that coadministration with itraconazole may increase the oral bioavailability of ZX-7101A, reduce bile excretion of ZX-7101, and consequently increase systemic exposure of ZX-7101. The results of this study indicate that the increase in exposure of ZX-7101 after combination with itraconazole is less than the exposure of ZX-7101 after a single oral dose of 80 mg ZX-7101A. According to the results of Phase I clinical safety studies and Phase II/III clinical safety and efficacy studies (Wu et al., 2024; Wang et al., 2025), there is no need to adjust the dose of ZX-7101A when combined with itraconazole. However, given the current absence of a standardized classification of P-gp inhibition strength, these findings cannot be generalized to all P-gp inhibitors.

Moreover, itraconazole coadministration did not alter the T_max_ of ZX-7101 in this study. Several reasons may account for this observation. ZX-7101A is a prodrug that undergoes rapid enzymatic conversion to ZX-7101, primarily mediated by carboxylesterases CES1-b, CES1-c, and CES2, while CYP450 enzymes are not involved. Given this metabolic pathway, the enzymatic biotransformation rather than intestinal transport may represent the rate-limiting step governing the appearance of ZX-7101 in plasma, which may explain why itraconazole did not alter the T_max_ of ZX-7101 despite its known inhibitory effect on P-gp. Preclinical Caco-2 studies demonstrated pronounced P-gp–mediated efflux for both ZX-7101A and ZX-7101. However, the absence of a meaningful *in vivo* effect of itraconazole on absorption rate parameters suggests that P-gp-mediated efflux may not be the primary determinant of early absorption in humans, consistent with previous reports indicating that modulation of P-gp activity predominantly affects the extent rather than the rate of absorption for many orally administered substrates (Elmeliegy et al., 2020). Factors such as passive permeability, rapid dissolution, and CES-mediated metabolic conversion may contribute to this observation. Clinically, understanding the metabolic and transporter pathways of ZX-7101A is essential for anticipating potential drug–drug interactions and identifying patient populations in whom altered enzyme activity, organ impairment, or concomitant medications may influence systemic exposure. Further mechanistic and clinical evaluations, particularly in patient populations or under repeated dosing, will be required to fully characterize the transporter and metabolic contributions to ZX-7101A disposition even though the current study demonstrates no significant interaction with itraconazole.

In the present study, one subject experienced creatine kinase elevation during the follow-up period after ZX-7101A/itraconazole coadministration, which was a CTCAE Grade 4 SAE with moderate severity. The AE was reported as a SAE on D62. This SAE was resolved completely after 18 days of active treatment with hydration and alkalization. Potential contributing factors, such as physical exercise or concomitant use of other drugs, were excluded. The creative kinase elevation occurred 20 days after a single dose of ZX-7101A in Stage 2, without any abnormal increase in plasma concentration of ZX-7101 in this subject. Furthermore, although creatine kinase levels were elevated, the plasma concentration of ZX-7101was very low on D20 after taking 40 mg ZX-7101A tablets. Based on these findings, it was concluded that this SAE was unlikely related to ZX-7101A but possibly associated with the use of itraconazole. It has been reported that treatment with ketoconazole, itraconazole, or voriconazole can induce myositis and rhabdomyolysis (Ferrari et al., 2004; Shanmugam et al., 2009). Patients in these cases usually presented with fatigue, extensive muscle pain, and elevated creatine kinase (ranging from 379 to 5200 IU/L) (Benitez and Carver, 2019). Considering the above literature reports, and the limitations of this study that the plasma concentration of itraconazole was not measured, it was impossible to further clarify the exact cause of creatine kinase elevation.

This study has several limitations that should be acknowledged. First, the study was conducted exclusively in healthy adult volunteers. This population provides a controlled environment for characterizing intrinsic pharmacokinetics and assessing drug–drug interaction potential. However, the findings may not fully translate to patients with comorbidities or to the intended disease population. Physiological alterations, concomitant medications, and organ impairment in clinical settings may influence the disposition of ZX-7101A or modify its interaction potential. Future studies in patient populations will therefore be essential to confirm the generalizability of the present results. Second, the overall sample size is modest, but consistent with the design of DDI studies. Furthermore, the precision of key PK parameters, together with the controlled study conditions, indicates that the number of participants was adequate for detecting potential interaction signals with itraconazole. Nevertheless, larger studies with more diverse participants will be required to further verify the robustness of the PK characteristics observed here.

## 5 Conclusion

Coadministration with itraconazole did not affect the T_max_ of ZX-7101, but increased C_max_, AUC_0-t_, and AUC_0-inf_ by 29.5%, 53.7%, and 58.0%, respectively. Additionally, itraconazole prolonged the T_1/2_ of ZX-7101 slightly by 17.6%. Concomitant use of multiple doses of itraconazole capsules increased the exposure of ZX-7101 after a single dose of 40 mg ZX-7101A tablets but the elevated exposure of ZX-7101 was still lower than the exposure of ZX-7101 after a single oral dose of 80 mg ZX-7101A alone. The results of a Phase II/III study demonstrated that a single dose of ZX-7101A 40 mg or 80 mg was effective in treating uncomplicated influenza in adults with good safety profiles (Wang et al., 2025). Therefore, there is no need to adjust the dose of 40 mg ZX-7101A when coadministered with itraconazole. The overall safety and tolerability of ZX-7101A are good in healthy subjects.

## Data Availability

The raw data supporting the conclusions of this article will be made available by the authors, without undue reservation.
